# A Novel Tensile Fracture Location of Friction Plug Welding (FPW) Joints

**DOI:** 10.3390/ma17235814

**Published:** 2024-11-27

**Authors:** Yu-Shu Wang, Xue-Qi Lv, Chun-Ming Xia, Xiong-Ying Li, Jie Yang, Chong-Gui Li

**Affiliations:** 1 School of Materials Engineering, Shanghai University of Engineering Science, Shanghai 201620, China; wangyushu997@163.com (Y.-S.W.);; 2 School of Mechanical and Automotive Engineering, Shanghai University of Engineering Science, Shanghai 201620, China; 3 College of Energy Engineering, Zhejiang University, Hangzhou 310007, China

**Keywords:** deformed plug center zone, tilt fiber-like microstructure, friction plug welding, shear fracture, mechanical properties

## Abstract

The fracture position of a friction plug welding (FPW) joint is typically located at or near the thermo-mechanically affected zone (TMAZ). Here, we found that microcracks in all FPW specimens initiate at the deformed plug center (DPC) zone and then propagate through the plug center along 45° shear surfaces, because the lowest hardness occurs at the DPC zone rather than the TMAZ or other zones, and the DPC zone presents a tilt fiber-like microstructure. Such a tilt microstructure stimulates formations and deformations of microvoids and propagation of microcracks along 45° shear surfaces. The ultimate tensile strength (237.7 MPa) and yield strength (220.8 MPa) of the FPW joint reach 78.8% and 85.7% of the base metal, respectively. These results indicate that 6061-T6 aluminum alloy can be effectively joined by the FPW technique.

## 1. Introduction

Lightweight aluminum (Al) alloys have been widely used in the aerospace field [1], but there exist several problems, such as the formation of spot defects during the friction stir welding process, the formation of keyhole defects after welding [2], and the repair of fastener holes with fatigue damage in aerospace structures [3]. These issues can be effectively solved by the friction plug welding (FPW) technique [4,5,6]. The mechanism of FPW is solid-phase repair: defects are first removed to form plug holes which are subsequently filled by high-speed rotating plug metals that are in the sizes matching to plug holes; a brake of the rotating plug begins when the joining interface reaches a plastic state via heat and forces generated from the relative rotation between the plug metal (PM) and the plug hole, until the cooling of joints. By this technique, the tensile strength and the fatigue life of welded structures can be improved via reducing residual stress and the burning loss of alloy elements [7,8].

Macro- and micro-structures of FPW joints are nonuniform, because the special geometric shapes of plugs and plug holes as well as the complicated reaction between heat and forces result in nonuniform distributions of temperature and stress [9]. The FPW joint of 6082 Al alloy was classified into four zones in the experiment from Zhao et al. [10], owing to the distinct fluidity of the plastic metal. The experiment of the FPW joint for 6082-T6 Al alloy by electron backscatter diffraction (EBSD) from Li et al. [11,12] illustrated that the preferred orientations of grains are distinct in different regions, leading to the formation of textures, and the thermo-mechanically affected zone (TMAZ) exhibits the largest size of grain and the smallest proportion of large-angle grain boundaries.

The structural inhomogeneity of FPW joints is difficultly eliminated by heat treatment and, more importantly, deteriorates the mechanical properties to certain extents. The experiment of the friction plug pull welding (FPPW) of 18-mm-thick AA2219-T87 plates from Liang et al. [13] reported that the bonding mechanism of the joint is the dynamic recrystallization of the Al alloy at and near the bonding interface, while the insufficient dynamic recrystallization at the top and bottom of the joint can cause the formation of kissing-bond defects that are the sources of microcracks. The experiment from Metz et al. [8], concerning the extruded and elongated grain structures or configurations at fracture sites in the FPW joints of 2195 Al-Li plates welded by friction stir weld, reported the occurrence of fractures in and near the TMAZ. The experiment from Gao et al. [14], concerning welding configurations and mechanical properties of AA-5A06 FPPW joints, demonstrated that the softening of the TMAZ and the occurrence of fractures at this zone result from the formation of coarse grains and the aggregation of secondary phases that continuously arrange along a certain direction. The softening of TMAZ and the weakened performance of joints, in the FPPW study of precipitate evolution of TMAZ for the TIG-weld 2219-T87 specimens from Shao et al. [15], are attributed to the dissolution of the θ’ phase and the existence of the large-size θ phase. These phases were extensively observed in FPW joints, including the TMAZ in the PM and base metal (BM) zones, the recrystallized plug metal (RPM) zone, and the heat-affected zone (HAZ) [16,17].

Considerable investigations have focused on the softening and structural inhomogeneity of the TMAZ and its effect on mechanical properties of FPW joints, because fracture positions are typically located at the TMAZ. Recently, Zhang et al. [6] reported the fracture position at the bonding interface between the base metal (BM) and the PM. Here, microcracks are observed to initiate at a different region—the deformed plug center (DPC) zone—and propagate through the plug center along 45° shear surfaces, possibly implying the structural inhomogeneity of the plug center. However, studies focusing on this aspect have been hardly reported. Why is the structure of the plug center nonuniform? Why do microcracks initiate at the DPC zone rather than the TMAZ? How does rotation speed affect the macro- and micro-structural inhomogeneity and mechanical properties of the FPW joint? These issues inspire us to conduct FPW experiments of the macro- and micro-structures and the mechanical properties of the joints for 6061-T6 Al alloy.

## 2. Experimental Materials and Methods

FPW processes were performed by using the welding equipment provided by Guohan Welding (Shanghai) Intelligent Technology Co., Ltd. (Shanghai, China). The BM and the PM are 6061-T6 Al alloy, both with identical chemical compositions and mechanical properties (Table 1).

**Figure 1:**
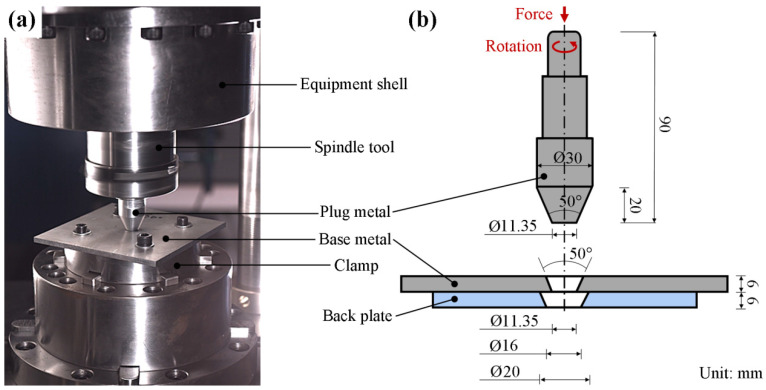
Assembly diagram and critical sizes. (**a**) Assembly relationships among plug metal, base metal and back plate, and (**b**) assembly diagram.

Before welding, the plug, the base plate, and the back plate were machined into selected shapes and sizes and then fixed to the clamp of the equipment in the assembly relationship given in Figure 1a. The base plate is in a dimension of 150 × 150 × 6 mm³. The plug and the plug hole are both in a bottom diameter of 11.35 mm and a taper angle of 50°. Other key sizes are given in Figure 1b. As the FPW process starts, the plug is pressed down by an upset force of 35 kN and in a pushing amount of 6 mm. The rotating speeds of the plug are set as 4000, 4500, and 5000 rpm.

The cross sections of the welded specimens were prepared by manual grinding and careful polishing. Prepared surfaces were etched with 0.5 mL HF and 100 mL distilled water for about 60 s. Microstructure analysis of welded specimens is performed using a Keyence VHX-600K optical microscope (Osaka, Japan) and an S-3400N scanning electron microscope (SEM) equipped with energy-dispersive X-ray spectroscopy (Tokyo, Japan).

Vickers microhardness measurement was performed by a Vickers indenter (HXD-1000TMSC/LCD, Shanghai, China) at a load of 0.98 N and dwell period 15 s. Microhardness was measured along the horizontal direction through location with a depth 3 mm from the upper surface of the FPW joint.

**Figure 2:**
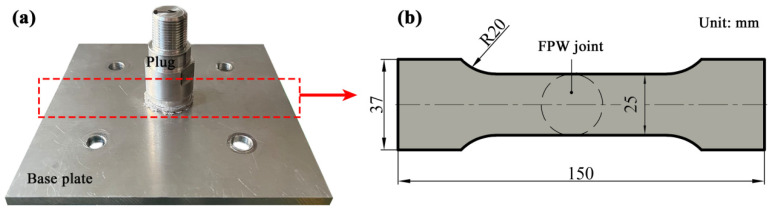
Configuration of (**a**) the welded specimen and (**b**) the tensile specimen.

Tensile tests were carried out by using a tensile testing machine (Zwick/Rollell Z5KN, Ulm, Germany) at a constant displacement rate of 1 mm/min at room temperature. The schematic of tensile specimen is shown in Figure 2. For each given rotation speed, a minimum of three tests were conducted. The yield strength (at 0.2% residual plastic strain), the ultimate tensile strength and the elongation are an average over three specimens.

## 3. Results and Discussion

### 3.1. Macroscopic Forming and Material Flow

**Figure 3:**
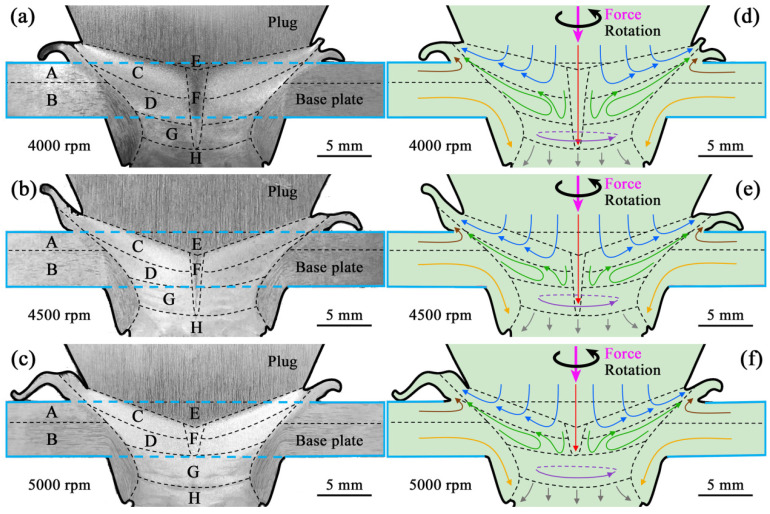
The FPW joints of 6061-T6 Al alloy: (**a**–**c**) macrostructural morphology and (**d**–**f**) corresponding material flow during the FPW process.

Figure 3 presents the macrostructure and corresponding material flow behavior of the FPW joints on the rotation-speed scale of 4000–5000 rpm. Integrally, each FPW joint (marked by the pale blue rectangle) exhibits a well-formed macrostructure because of the sufficient material flow in the joint, as indicated by the defect-free bonding interface and the uniform macrostructures of the top and bottom flashes.

For different regions, macrostructures are distinct at a certain rotation speed owing to different material flow behaviors. Based on this, the FPW joint can be divided into regions A–H by the black dashed lines shown in Figure 3. In region A, which is located at the upper base plate but outside of the bonding interface, material is squeezed and becomes soft when the plug contacts the base plate. The softened plastic material flows upward and is extruded to form the top flash, because this region is free of external constraint. Such a region is referred to as the top flash formation zone. Below region A, region B is subjected to heat and downward force and exhibits downward material flow, referred to as the lower deformed plate zone.

In region C, a zone between region A and the center region of the joint, complicated material reflow is observed, including the downward flow resulted from the upset force, the circumferential flow induced by the rotation of the plug, and the upward flow resulted from the hindering effect of the lower region. Such a region is referred to as the upper reflow zone. Oppositely, region D is the lower reflow zone, which is right below region C.

Region E, which is located at the top and the center of the FPW joint, exhibits downward material flow, indicated by the vertical rolled morphology of macrostructure, referred to as the upper downward flow zone. Right below region E is region F, which also exhibits downward material flow but is smaller than region E, because of the strong circumferential material flow at the top. Thus, region F is referred to as the lower downward flow zone.

Region G exhibits circumferential material flow indicated by the circle macrostructural morphology, referred to as the bottom circumferential flow zone. The circumferential material flow is stronger here than in other zones, because region G is the zone that first contacts the rotating plug and material softening is more remarkable than in the reflow zones, promoting material flow. Region H, which is located at the bottom of the plug and right below region G, displays downward material flow into the hole of the back plate.

As the rotation speed varies from 4000 to 5000 rpm, the macrostructure of the FPW joint significantly changes with the gradually enhanced circumferential material flow. Regions C and D become smaller as regions E and region G become larger. Region F is wide and deep into the bottom of region G at 4000 rpm but becomes narrow at 4500 rpm. At a faster rotation speed of 5000 rpm, the upper part of region F becomes wider, and the lower part disappears, owing to the more sufficient circumferential material flow. Moreover, region F exhibits a macroscopic morphology as those of regions C and D, possibly because the region F at 5000 rpm consists of dynamically recrystallized structures as those of the regions C and D, due to high welding heat and strong circumferential material flow. The evolution of region F indicates that the macrostructural inhomogeneity of this region becomes weaker at large rotation speeds, and is even eliminated at 5000 rpm.

### 3.2. Microstructure

**Figure 4:**
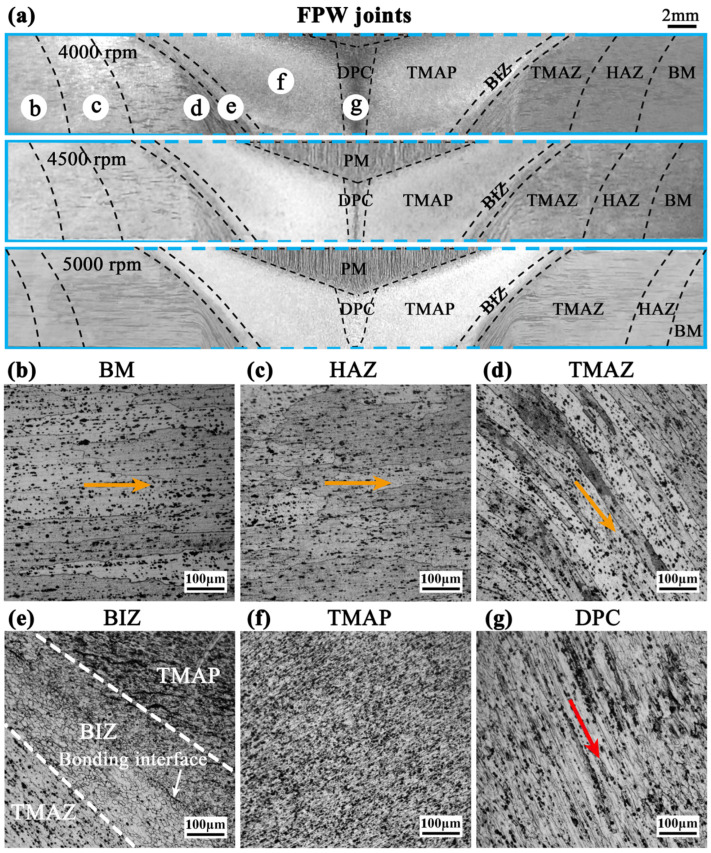
Microstructural morphology of the FPW joints. The yellow and red arrows mark the orientations of deformed grains.

Based on the metallographic results, the microstructure of each FPW joint can be distinguished as seven zones from the outside to the center: BM, HAZ, TMAZ, bonding interface zone (BIZ), thermo-mechanically affected plug (TMAP), PM, and deformed plug center (DPC), as shown in Figure 4.

In the BM zone, grains still exhibit horizontal-strip configurations after rolling, because this zone is far from the BIZ and thus hardly affected by frictional heat and pressure (Figure 4b). In the inner zone, HAZ, grains still remain in horizontal-strip configurations as in the BM zone but become larger, indicating that this zone is affected by friction heat but free of the downward force (Figure 4c). In the TMAZ, grains are visibly stretched to form more narrow strips and steadily deviate from the initially horizontal direction to the downward direction in the tilt angles depending on their positions, owing to the downward force and the confinement of the plug hole (Figure 4d). From the BM zone to the TMAZ, the orientation evolution (marked by the yellow arrows) of microstructures is consistent with the direction of material flow in the region B (Figure 3). As the rotation speed varies from 4000 to 5000 rpm, the HAZ moves to a more outside position, and the TMAZ becomes larger (Figure 4a), indicating the larger cross-section size of the entire FPW joint.

In the BIZ, strip-like grains transfer into distortionless equiaxial grains (Figure 4e), leading to the initial contact surface between the plug and the base plate changing into a well-bonded bonding interface. Similar grain refinement is also observed in the TMAP zone, which is free of coarse grains (Figure 4f). The complex combining effect of frictional heat, upset force, and rotation in these two zones makes material flow more sufficient, readily enhancing the anisotropy of grains. Based on the fractional calculus approach to describing the dynamics of complex energy transfer phenomena [18,19], anisotropic grains in the nanoscale size can enhance the transport of frictional heat, leading to the occurrence of dynamic recrystallization. Hence, the grains in the BIZ and TMAP zones are remarkably smaller than that in the BM zone (Figure 4e).

The microstructure of the plug center in the FPW joint is nonuniform, including two zones: the upper PM and the lower DPC. The orientations of microstructures of these two zones are distinct. In the PM zone, the microstructure is similar to that of the BM zone after rolling, but grains exhibit vertical-strip configurations. In the DPC zone, the microstructure is dramatically stretched to form a fiber-like configuration by the upset force (Figure 4g). However, the orientation of the fiber-like microstructure is not parallel to but deviates from the vertical-strip microstructure of the PM zone (or from the upset force) in a certain tilt angle (marked by the red arrow), due to deformations of grains induced by the circle force from the rotating plug. Such a tilt fiber-like microstructure induces the arrangement and aggregation of secondary phase particles in the tilt-angle direction.

**Figure 5:**
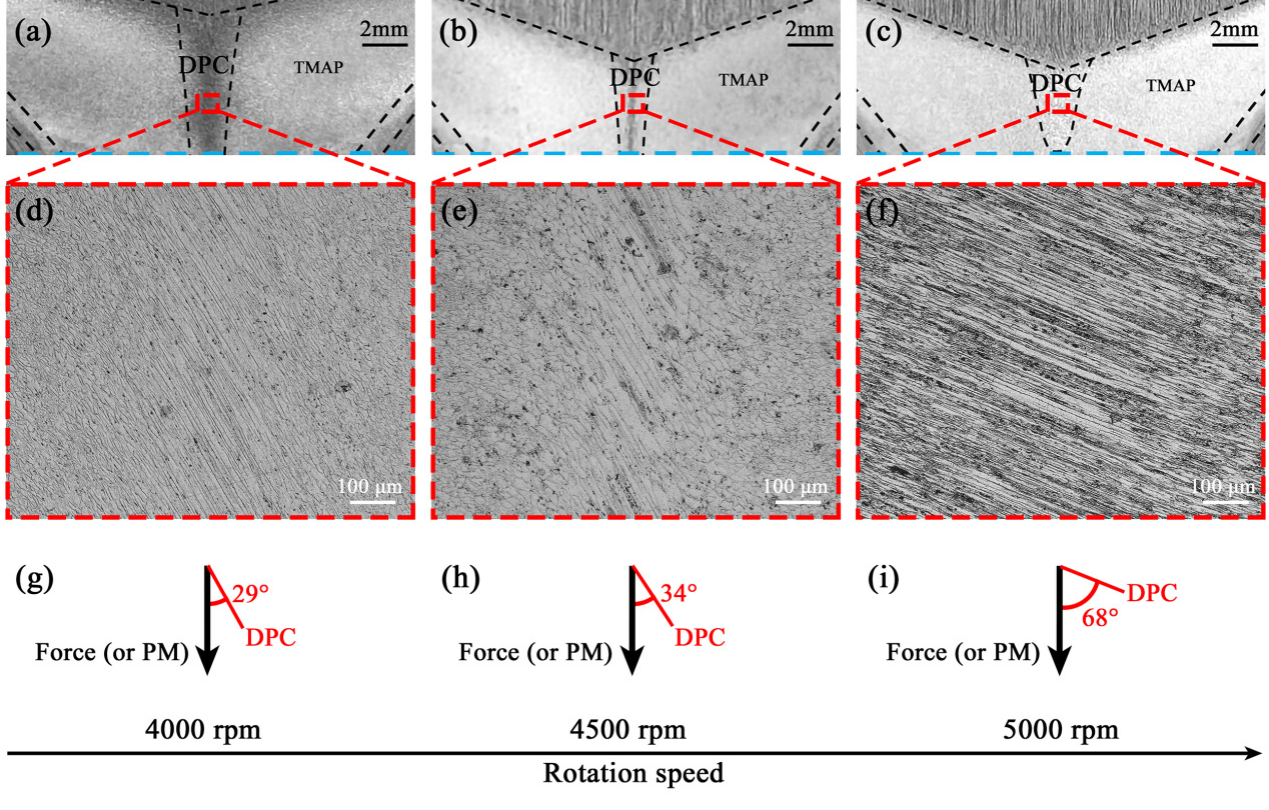
Structural morphology of the DPC zones in the FPW joints. (**a**–**c**) macrostructures, (**d**–**f**) microstructures, and (**g**–**i**) tilt angles of microstructures.

For the DPC zone, although macrostructural inhomogeneity becomes weaker at large rotation speeds and even is eliminated at 5000 rpm (Figure 5a–c), microstructure is still nonuniform at all rotation speeds, as indicated by the central tilt-orientation microstructure that significantly differs from those of the surrounding PM and TMAP zones (Figure 5d–f). These different orientations among the microstructures of the DPC, PM, and TMAP zones would induce stress concentration at the plug center during a tensile test. Moreover, the stress concentration possibly becomes stronger as the rotation speed increases from 4000 to 5000 rpm, because (i) the PM zone and the boundary between the PM zone and other zones significantly increase and (ii) the tilt angle of the microstructure in the DPC zone increases from 29° at 4000 rpm to 34° at 4500 rpm and then to 68° at 5000 rpm, owing to the enhanced rotation and circumferential material flow (Figure 5g–i).

Besides, Figure 5d–f shows that the microstructure of the DPC zone at 4000 and 4500 rpm consist of dynamically recovered grains, as indicated by the coarse grains that are subsequently stretched. While at 5000 rpm, grains become smaller, and a large amount of secondary phase particles are observed. Thus, the microstructure of the DPC zone at 5000 rpm mainly consists of dynamically recrystallized grains as those of the TMAP zone, because of higher welding heat and a larger rotation force. This is the reason why the macrostructural morphology of the DPC zone is similar to that of the TMAP zone.

### 3.3. Mechanical Properties

#### 3.3.1. Hardness Distribution

**Figure 6:**
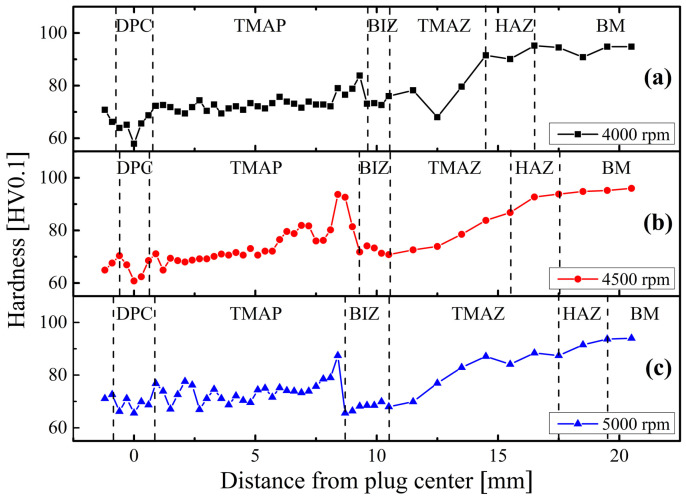
Hardness distributions of the FPW joints at (**a**) 4000 rpm, (**b**) 4500 rpm and (**c**) 5000 rpm.

The inhomogeneous macro- and micro-structures lead to the nonuniform hardness distributions on the cross-section surfaces of the FPW joints, as shown Figure 6. Integrally, the hardness distribution presents a remarkable decreasing tendency from the BM zone to the BIZ, a small peak just exceeding the BIZ and then a slightly decreasing tendency from the outside TMAP zone to the center of the DPC zone. The valley values of hardness occur in the BIZ and DPC zones rather than the TMAZ, indicating that (i) the material softening in these two zones is more severe than in the TMAZ and (ii) microcracks possibly initiate at these two zones.

The hardness of the precipitation-strengthened 6061 Al alloy mainly depends on the sizes of grains, especially precipitated phases. Therefore, the occurrence of grain coarsening as well as the dissolution and aggregation of secondary phase particles leads to lower hardness values in the HAZ zone in comparison with those of the BM zone. In the TMAZ and BIZ that are subjected to abundant friction heat and large stirring forces, although grains become finer, the dissolution and aggregation of the large amount of secondary phase particles eventually lead to the further decrease in hardness. Partial secondary phases in the BIZ redissolve into the outside TMAP zone, and precipitate in a dispersed form after absorbing the solid solution elements, resulting in the small peak in hardness. The hardness of the other TMAP regions gradually decreases because of the coarsening of secondary phases. Except for the coarsening of secondary phases, the coarser grains also lead to the lower hardness of the DPC zone in comparison to the TMAP zone.

At 5000 rpm, the hardness of the DPC zone is quite close to that of the BIZ, indicating that the DPC zone mainly consists of dynamically recrystallized microstructure similar to that of the BIZ. While at 4000 and 4500 rpm, the hardness of the DPC zone is lower than that of the BIZ, as well as than that of the DPC zone at 5000 rpm, indicating that the DPC zones at such conditions exhibit dynamically recovered microstructures rather than the one at 5000 rpm.

#### 3.3.2. Tensile Property

**Figure 7:**
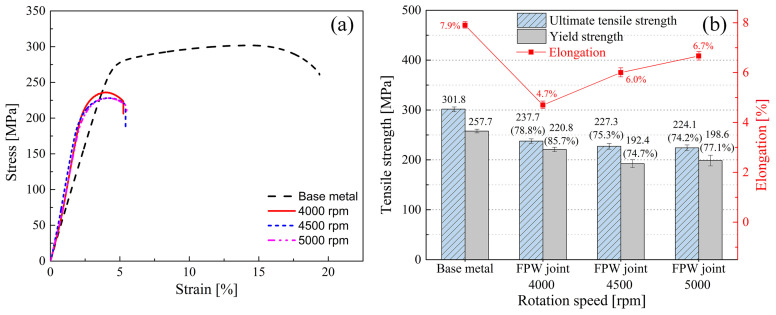
Tensile property of the FPW joints. (**a**) The stress VS. strain, and (**b**) tensile strength.

Figure 7 shows the tensile property of the base metal and of the FPW joints at all of the rotation speeds. The base metal exhibits an ultimate tensile strength of 301.8 MPa, a yield strength of 257.7 MPa, and an elongation of 7.9%. The ultimate tensile strength of the FPW joint gradually decreases from 237.7 MPa at 4000 rpm to 224.1 MPa at 5000 rpm, which are 78.8% and 74.2% of the base metal, respectively. In contrast, the elongation of the FPW joint increases from 4.7% at 4000 rpm to 6.7% at 5000 rpm. The lowest yield strength of the FPW joint is 192.4 MPa (74.7% of the base metal) at 4500 rpm, and the highest yield strength of the FPW joint is 220.8 MPa (85.7% of the base metal) at 4000 rpm. The confused point here is that, at 4000 rpm, the hardness of the DPC zone is the lowest (Figure 6), but the ultimate tensile strength of the FPW joint is the highest, which will be discussed later.

**Figure 8:**
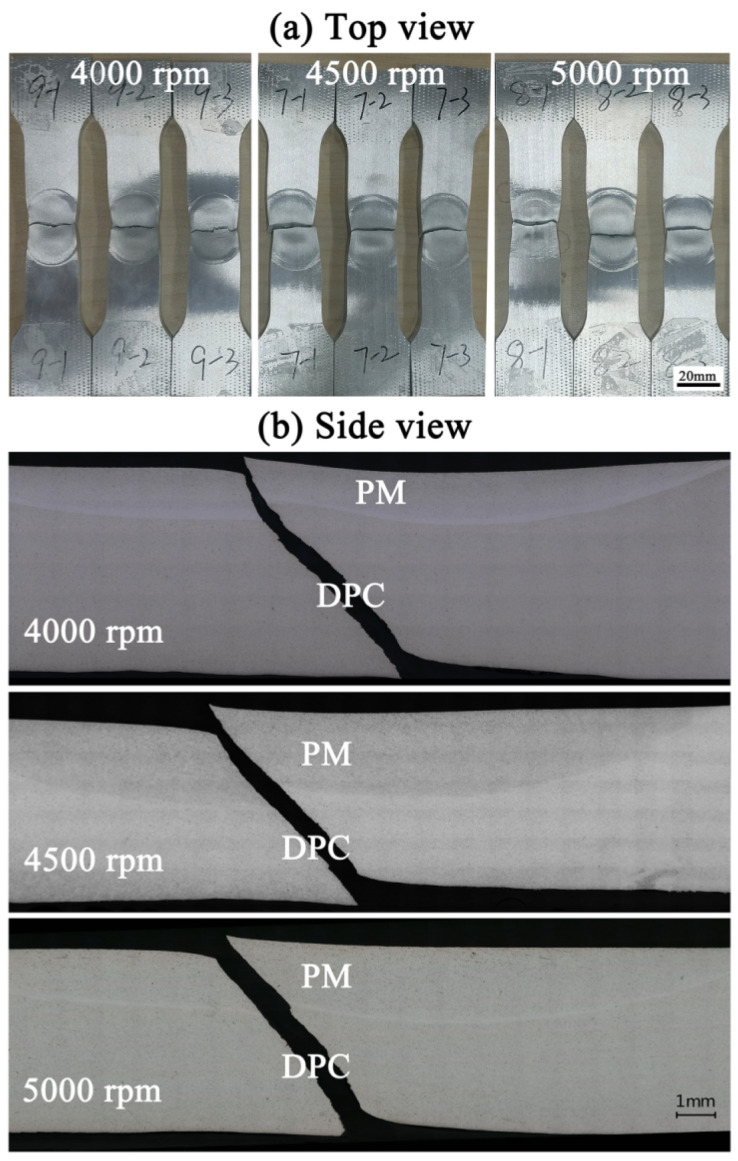
Tensile fractures of the FPW joints: (**a**) top view and (**b**) side view.

Figure 8 shows the top and side views of the FPW specimen after the tensile test. Necking is observed in all specimens, indicating the good plasticity of the FPW joints. At necking, there exists a visible boundary between the upper PM zone and the surrounding DPC and TMAP zones, indicating the macrostructural inhomogeneity of the plug center. Fractures of all specimens are located at the plug center rather than other zones, because both the macro- and micro-structures of the plug center are nonuniform (Figure 3, Figure 4 and Figure 5), leading to stress concentration. The PM zone was dramatically stretched, suggesting that (i) the PM zone exhibits high plasticity and (ii) microcracks initiate at the DPC zone.

Microcracks initiate at the DPC zone and propagate along 45° shear surfaces, mainly because of its tilt fiber-like microstructure and severe material softening. Microcracks of the FPW joint prefer to initiate at the zone where material is dramatically extruded and stretched [8] or material softening is severe [14,15,16,17], such as the TMAZ and BIZ [6]. Here, compared to the PM zone where grains were stretched vertically downward and to the BIZ zone that also presents a hardness valley but is free of special-orientation grains, the secondary phase particles that arrange and aggregate along the tilt fiber-like microstructure in the DPC zone (Figure 5) readily induce the occurrence, growth, and deformation of microvoids along 45° shear surfaces when the specimen is subjected to shear forces during the tensile process, leading to the formation of microcracks at the DPC zone and the propagation of these microcracks to the entire plug center along 45° shear surfaces. Compared to the TMAZ that also exhibits a tilt-orientation microstructure, the material softening is more severe in the DPC zone than in the TMAZ (Figure 6). Thus, microcracks prefer to initiate at the DPC zone rather than the TMAZ or other zones.

Although all of the tensile specimens present a shear fracture at the plug center, the mechanical properties of the FPW joints vary with the increasing rotation speed. The factors that should be taken into consideration are as follows: (i) the microstructure of the DPC zone, (ii) the tilt angle of the microstructure of the DPC zone, and (iii) the size of the PM. As the rotation speed increases from 4000 to 5000 rpm, although the microstructure changes from the coarse dynamically recovered grains to the dynamically recrystallized grains (Figure 5), stress concentration in the plug center becomes more severe, because the sizes of the PM zone and the boundary between the PM and the surrounding TMAP and DPC zones as well as the tilt angle of the microstructure in the DPC zone become larger (Figure 5 and Figure 8). This is the reason why the DPC exhibits the lowest hardness values, but the FPW joint still presents the highest tensile strength at 4000 rpm.

**Figure 9:**
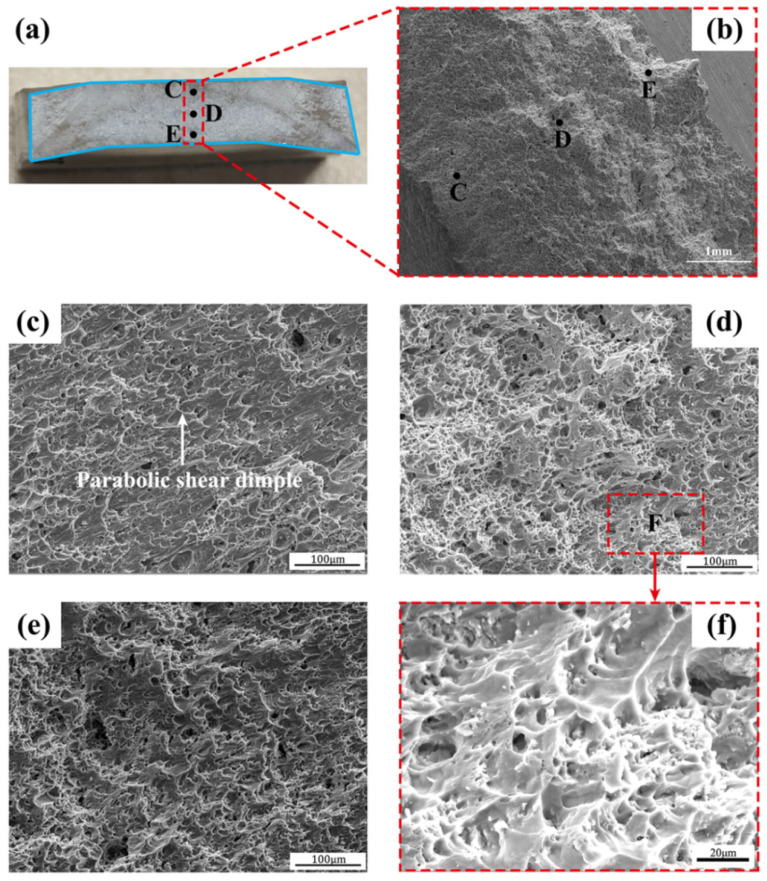
Fracture configurations of the FPW joint at 4000 rpm (right view of Figure 8b). (**a**,**b**) fracture surface. The upper, middle and lower zones at the center of the fracture surface are marked by C, D and E, respectively. (**c**–**f**) micro configurations at the zones C–F, respectively.

Figure 9 shows fracture configurations of the FPW joints at 4000 rpm, obtained from the right view of Figure by SEM. A tensile fracture surface is generally classified as three zones: the central fibrous zone that consists of equiaxial dimples, the external shear lip zone that consists of parabolic shear dimples, and the middle radial zone. Here, the fracture in region C consists of elongated parabolic shear dimples (Figure 9c), exhibiting a fracture characteristic of the external shear lip zone. The fracture in regions D and E mainly consist of elongated parabolic shear dimples except for a few equiaxial dimples (Figure 9d–f), indicating that region D presents a shear fracture characteristic as that of the shear lip zone, even though it is a fibrous zone. Thus, microcracks initiate at the DPC zone and then propagate to the PM and TMAP zone. This is mainly because the structural orientation of region D deviates from that of the PM zone in a certain tile angle (Figure 5). Another possible reason for the shear fracture at the DPC zone is that this fibrous zone is too small because the specimen is too thin and its thickness is far smaller than its width (Figure 2 and Figure 3), leading to the shear fracture characteristic of the shear lip zone. There is not direct evidence for the relationship between the novel fraction position and the cross-section size of the entire FPW joint, although this size significantly increases with the increasing rotation speed.

## 4. Conclusions

Experimental studies of 6061-T6 Al alloy were conducted to obtain defect-free FPW joints at 4000–5000 rpm. Macro- and micro-structures, material flow, mechanical properties, and fracture behavior of the FPW joints were investigated. Main conclusions are as follows:

(1) 6061-T6 aluminum alloy can be effectively joined by the FPW technique, as indicated by the defect-free macrostructure, fracture positions of all joints at the plug center, and 78.8% (or 85.7%) of the ultimate tensile strength (or yield strength) of the base metal at 4000 rpm.
(2) The marco- and micro-structures of the FPW joint are nonuniform. At the plug center, macrostructural inhomogeneity can be improved via increasing rotation speed, but microstructures at all of the selected rotation speeds are still uniform and consist of the upper plug metal (PM) zone and the lower deformed plug center (DPC) zone, leading to stress concentration and fractures at this region.
(3) Microcracks initiate at the DPC zone rather than the widely reported thermo-mechanically affected zone (TMAZ) or other zones, because the formation of the tilt fiber-like microstructure and the severe material softening in the DPC zone lead to the initiation of microcracks at this zone and facilitate the propagation of microcracks along 45° shear surfaces.

These results indicate that 6061-T6 aluminum alloy can be effectively joined by the FPW technique, especially at the rotation speed of 4000 rpm. FPW experiments of 6061-T6 Al alloys with the plug hole in different sizes and on a wider scale of rotation speeds should be performed in future studies to verify the novel fracture position.

## Figures and Tables

**Table 1:** Chemical compositions and mechanical properties of 6061-T6 Al alloy.

Chemical Compositions									
Element	Mg	Si	Fe	Cu	Mn	Cr	Zn	Ti	Al
Standard value [%]	1.15	0.64	0.59	0.25	0.13	0.1	0.15	0.02	Balance
**Mechanical Properties**									
Yield strength, R_p_0.2 [MPa]			257.7		Elogation percentage [%]				7.9
Ultimate tensile strength [MPa]			301.8		Hardness [HV_0.1_]				97

## Data Availability

The original contributions presented in this study are included in the article. Further inquiries can be directed to the corresponding author.
